# Clinical Outcomes and Adverse Events of Endoscopic Submucosal Dissection for Gastric Tube Cancer after Esophagectomy

**DOI:** 10.1155/2019/2836860

**Published:** 2019-03-03

**Authors:** Ko Watanabe, Takuto Hikichi, Jun Nakamura, Minami Hashimoto, Tadayuki Takagi, Rei Suzuki, Mitsuru Sugimoto, Hitomi Kikuchi, Naoki Konno, Mika Takasumi, Yuki Sato, Hiroki Irie, Katsutoshi Obara, Hiromasa Ohira

**Affiliations:** ^1^Department of Endoscopy, Fukushima Medical University Hospital, 1 Hikarigaoka, Fukushima, Japan; ^2^Department of Gastroenterology, Fukushima Medical University School of Medicine, 1 Hikarigaoka, Fukushima, Japan; ^3^Department of Advanced Gastrointestinal Endoscopy, Fukushima Medical University, 1 Hikarigaoka, Fukushima, Japan

## Abstract

**Background and Aim:**

The clinical outcomes of endoscopic submucosal dissection (ESD) for gastric tube cancer (GTC) after esophagectomy remain unclear. The aim of this study was to evaluate the clinical outcomes and safety of ESD for GTC.

**Patients and Methods:**

Twenty GTC lesions in 18 consecutive patients who underwent ESD between February 2008 and June 2018 were included in this retrospective study. The endpoints were the treatment outcomes of ESD (i.e., *en bloc* resection rate, complete *en bloc* resection rate, and curative resection rate), the adverse events following ESD, and the long-term outcomes.

**Results:**

The *en bloc* resection rate was 100%, while the complete *en bloc* resection rate and curative resection rate were 80% each. Adverse events were observed in 16.7% (3/18) of patients: one postoperative bleeding, 1 intraoperative perforation that required emergency surgery, and 1 pyothorax that required chest drainage. The 1-, 3-, and 5-year overall survival rates were 100%, 70.9%, and 70.9%, respectively. Although local recurrence was detected in 1 case of noncurative resection, no GTC- or ESD-related deaths were observed.

**Conclusion:**

ESD for GTC was feasible and acceptable to enable *en bloc* resection and to prevent cancer death. However, ESD for GTC should be performed more carefully than common gastric ESD because serious adverse events specific to the gastric tube may occur.

## 1. Introduction

Esophageal cancer is known to be associated with metachronous cancers in other organs, particularly head and neck cancer and gastric cancer [1–4]. Recently, the survival rate of esophageal cancer patients after esophagectomy has improved due to progress in surgical techniques and multidisciplinary therapies [5–8]. Therefore, esophagogastroduodenoscopy (EGD) procedures have been performed in esophageal cancer patients after esophagectomy; consequently, EGD has sometimes revealed gastric tube cancer (GTC) in the reconstructed gastric tube. In Japan, endoscopic submucosal dissection (ESD), which enables reliable *en bloc* resection as a minimally invasive treatment, is now a standard treatment for early gastric cancer (EGC), particularly for differentiated mucosal cancers that have a low risk of lymph node metastasis [9, 10]. However, we are concerned that ESD for GTC may be a technically difficult procedure because of the anatomical features of the gastric tube, such as its narrow working space, deformities, mucosal inflammation, severe fibrosis with staples under the suture line, and mediastinal operation. Several researchers have reported the benefit and safety of ESD for GTC [11–17]. However, we have experienced serious adverse events, including a pyothorax. Therefore, we conducted this study to evaluate the clinical outcomes and safety of ESD for GTC.

## 2. Patients and Methods

### 2.1. Patients

Twenty GTC lesions in 18 consecutive patients were treated by ESD at the Fukushima Medical University Hospital between February 2008 and June 2018. Procedural and clinical data were collected and analyzed retrospectively from a prospectively maintained endoscopy database.

The indications for ESD in EGC at our institution include the following, which are based on endoscopic diagnosis with mucosal biopsy: (1) differentiated intramucosal cancer without ulceration, (2) differentiated intramucosal cancer of 3 cm or less with ulceration, and (3) undifferentiated intramucosal cancer of 2 cm or less without ulceration. These indications are based on the Japan Gastric Cancer Treatment Guidelines [18]. These indications were similarly applied for GTC. All patients provided written informed consent before the ESD procedure, and this study was conducted with the approval of the Ethics Committee of Fukushima Medical University (approval no. 2407).

### 2.2. ESD Procedure

ESD was performed with a Dual Knife (KD-650L; Olympus Medical Systems Corp., Tokyo, Japan), a Flex Knife (KD-630L; Olympus Medical Systems Corp., Tokyo, Japan), an IT Knife 2 (KD-611L; Olympus Medical Systems Corp., Tokyo, Japan), or an SB Knife Jr (MD-47703; Sumitomo Bakelite, Tokyo, Japan). A single-channel endoscope (GIF-H260Z; Olympus Medical Systems Corp., Tokyo, Japan) was used to mark dots using magnifying narrow-band imaging, and another single-channel endoscope (GIF-Q260J; Olympus Medical Systems Corp., Tokyo, Japan) was used for mucosal incision and submucosal dissection. A 1 : 1 solution of 0.4% sodium hyaluronate (MucoUp; Johnson & Johnson K.K., Tokyo, Japan) and glycerol (Chugai Pharmaceutical Co. Ltd., Tokyo, Japan) was injected into the submucosa using a 25-G injection needle (ImpactFlow; TOP Corp., Tokyo, Japan) [19]. Hemostatic forceps (Coagrasper; FD410LR; Olympus Medical Systems Corp., Tokyo, Japan) were used for the prophylactic coagulation of blood vessels and hemostasis for intraoperative bleeding. A VIO300D or ICC200 (ERBE Elektromedizin GmbH, Tübingen, Germany) was used as the high-frequency generator. ESD was performed under sedation with a combination of midazolam (before February 2014) or propofol (after February 2014) and pentazocine in almost all cases. In contrast, in cases expected to be technically difficult, ESD was performed under general anesthesia with endotracheal intubation by an anesthesiologist. Patients fasted or maintained a low-residue diet starting the day before ESD because food residue is frequently seen in the gastric tube after esophagectomy. All ESDs were performed by expert physicians who were board-certified gastroenterological endoscopists of the Japan Gastroenterological Endoscopy Society and who had performed over 100 ESD procedures (T.H., K.W., and M.S.).

### 2.3. Outcomes

The endpoints were the treatment outcomes of ESD (i.e., the *en bloc* resection rate, complete *en bloc* resection rate, and curative resection rate), adverse events following ESD, and the clinical courses and long-term outcomes (i.e., overall survival rates, local recurrence in the lymph node and distant metastases, cause of death, and occurrence of metachronous GTC after ESD).

Pathological staging of the initial esophageal cancer was determined according to the Japanese classification of esophageal cancer established by the Japan Esophageal Society [20]. Pathological staging of the GTC was determined according to the Japanese classification of gastric carcinoma established by the Japanese Gastric Cancer Association [21]. The tumor locations were classified as upper, middle, or lower stomach, which were the same as in the unresected stomach. The resected specimens were sliced into 2 mm sections. Tumor size, histological type, depth of invasion, ulcer findings, lymphovascular invasion, and the horizontal and vertical resection margins were macroscopically assessed. The histological type was classified into the differentiated type (well- and moderately differentiated tubular adenocarcinomas and papillary adenocarcinomas) and the undifferentiated type (poorly differentiated adenocarcinomas, signet ring cell carcinomas, and mucinous adenocarcinomas). When the tumor exhibited a mixture of differentiated and undifferentiated types, the histological type was classified according to the majority of the tumor components. Complete *en bloc* resection was defined as resection of the tumor in a single piece that included the tumor-free margin. The specimens that satisfied the following criteria were considered to represent *en bloc* curative resections: (1) differentiated intramucosal cancer without ulceration and with no lymphovascular invasion, (2) differentiated intramucosal cancer of 3 cm or less with ulceration and with no lymphovascular invasion, (3) differentiated minimally invasive submucosal (invasion depth< 500 *μ*m from the muscularis mucosa: SM1) cancer of 3 cm or less without ulceration and with no lymphovascular invasion, and (4) undifferentiated intramucosal cancer of 2 cm or less without ulceration and with no lymphovascular invasion.

Regarding adverse events, perforation was diagnosed when the thoracic cavity was endoscopically visible or when free air was recognized on a computed tomography (CT) image. CT was performed only when a perforation might have occurred endoscopically during the ESD [19]. Postoperative bleeding was defined as the occurrence of hematemesis or the presence of tarry stool, with endoscopic confirmation of bleeding or exposed vessels. Aspiration pneumonia was diagnosed on radiography or if the patient experienced a fever of 38°C or higher. Stenosis was defined as the inability to pass the gastroscope, which had a diameter of 9.2 mm (GIF-Q260; Olympus Medical Systems Corp., Tokyo, Japan).

Follow-up endoscopic examinations were conducted two months after ESD and every 6 or 12 months thereafter [19]. CT was also performed every 3 or 6 months for patients with noncurative resections who were followed up without additional surgery and patients within five years from esophagectomy. Follow-up information was collected from the medical records. If the patients were followed up outside of our institution, we conducted a questionnaire survey with their primary care physicians. If the patient did not make any hospital visits during the follow-up period, we contacted their homes to determine whether they were alive.

### 2.4. Statistical Analysis

Values were reported as the medians with ranges. The overall patient survival rate was estimated using the Kaplan-Meier method. This analysis was performed using SPSS software (version 21 for Windows; IBM Corp, Armonk, NY, USA).

## 3. Results

### 3.1. Patient and Lesion Characteristics

The clinicopathological characteristics of the twenty GTC lesions in 18 patients are summarized in Table 1. The median age of the patients (17 men, 1 woman) was 72.5 years (range, 55-82 years). Synchronous GTCs were detected in two cases. All patients had undergone esophagectomy for esophageal cancer of the squamous cell carcinoma type. The median interval between the esophagectomy and ESD for GTC was 108 months (range, 24-264 months). For reconstruction, the retrosternal route was used in 10 patients, and the posterior mediastinal route was used in 8 patients. One lesion overlapped with a suture line of the previous surgery. No lesions on the anastomotic site of the previous surgery were included in this study. ESD was performed under general anesthesia for only 2 patients with large GTC with endoscopically suspected submucosal invasion and 1 patient with GTC adjacent to anastomosis. Food residue in the stomach was found in 4 patients (22.3%) in the endoscopic image that was obtained the day of ESD. The median resected specimen diameter was 36.5 mm (range, 23-76 mm), and the median tumor diameter was 16 mm (range, 8-61 mm).

### 3.2. Treatment Outcomes

The treatment outcomes are summarized in Table 2. The median procedure time was 87.5 min (range, 19-242 min). In 11 lesions, which were mainly located at the lower gastric tube, the procedure in retroflex position was not necessary during ESD. In the other 9 lesions, which were located at the upper and middle gastric tube, we attempted to perform ESD in both the retroflex and straight position. However, in 5 lesions, it was impossible to perform ESD in the retroflex position because of the narrow working spaces. The *en bloc* resection rate was 100%. However, the complete *en bloc* resection rate and the curative resection rate were each 80%. Of the patients who underwent noncurative resection, submucosal invasion to >500 *μ*M (SM2) with lymphovascular invasion and positive vertical margins was observed in 2 lesions. The other 2 patients who underwent noncurative resection were those with only positive horizontal margins (Table 3).

### 3.3. Adverse Events

**Figure 1:**
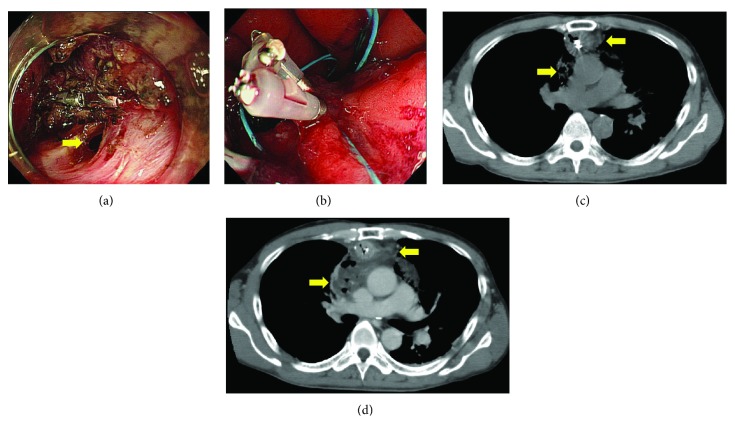
(a) An endoscopic image obtained during endoscopic submucosal dissection (ESD). Perforation occurred during the submucosal dissection (yellow arrow). (b) An endoscopic image obtained during ESD. Endoloops and endoclips were used in an attempt to close the perforation during ESD after the lesion was resected *en bloc*. (c) A computed tomography (CT) image taken immediately after ESD. CT revealed refluxed gastric and duodenal juice that leaked outside of the gastric tube (yellow arrow). (d) A CT image obtained the following day. CT revealed that the fluid had spread extensively within the mediastinum (yellow arrow), which led to the development of mediastinitis.

**Figure 2:**
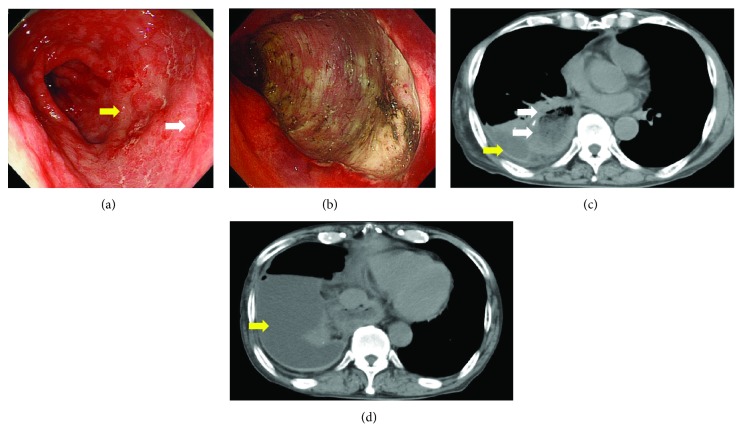
(a) An endoscopic image obtained during endoscopic submucosal dissection (ESD). White light imaging revealed 2 synchronous gastric tube cancers (yellow and white arrows) at the posterior wall of the lower gastric tube. (b) An endoscopic image obtained during ESD. Both lesions were resected *en bloc* in the same piece without perforation. (c) A computed tomography (CT) image obtained 2 days after ESD. CT revealed right pleural effusion (yellow arrow). The dilated and tortuous gastric tube in the posterior mediastinal reconstruction route markedly protruded into the right thoracic cavity, close to the pleura (white arrow). (d) A CT image obtained 3 weeks after ESD. CT revealed pyothorax of the right chest (yellow arrow).

Adverse events following ESD were observed in 3 cases (16.7%, Table 4). In one case, postoperative bleeding occurred 2 days after ESD, and emergency endoscopic hemostasis was performed successfully. In the other 2 cases, serious adverse events were reported, including intraoperative perforation that required emergency surgery and pyothorax that required chest drainage. In the case with intraoperative perforation, the lesion was located at the greater curvature of the middle gastric tube in the retrosternal reconstruction route; fibrosis of the submucosa was also observed. Endoloops and endoclips were used in an attempt to close the perforation [22] during ESD after the lesion was resected *en bloc* (Figure 1(a) and 1(b)). However, CT immediately after ESD revealed refluxed gastric and duodenal juice that leaked to the outside of the gastric tube (Figure 1(c)). Although antibiotics were administered and a nasogastric tube was inserted into the anal side of the ESD site, CT performed the following day revealed that the fluid had spread extensively into the mediastinum (Figure 1(d)) and revealed the development of mediastinitis. Therefore, this patient underwent emergency surgery. In the pyothorax case, two synchronous lesions were present at the posterior wall of the lower gastric tube in the posterior mediastinal reconstruction route (Figure 2(a)). Both lesions were resected *en bloc* in the same piece in 78 min without perforation (Figure 2(b)). However, a high fever developed after ESD, and CT performed 2 days after ESD revealed right pleural effusion (Figure 2(c)). Intravenous broad-spectrum antibiotics were administered for one week, the fever subsided, and a chest X-ray revealed a reduction of the pleural effusion. Therefore, this patient was discharged 9 days after ESD. However, he was hospitalized again because a high fever redeveloped 3 weeks after ESD. CT revealed a right pyothorax (Figure 2(d)). EGD did not reveal delayed perforation. Therefore, he underwent a percutaneous chest drainage, and antibiotics were maintained. The clinical signs of the patient gradually improved after the procedure, and he was discharged 6 weeks after chest drainage.

### 3.4. Clinical Courses and Long-Term Outcomes

**Figure 3:**
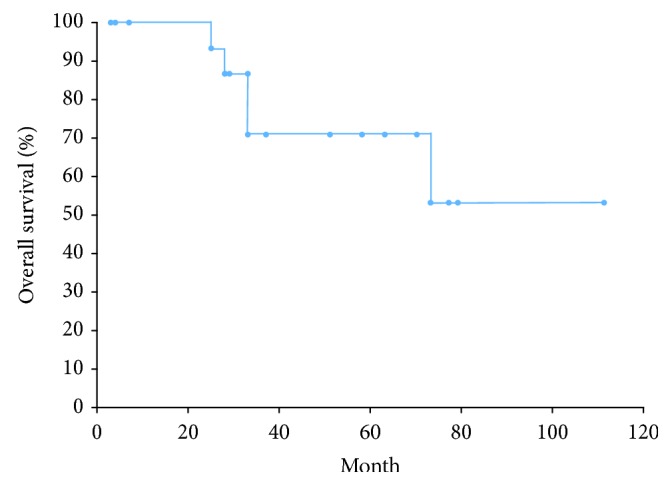
Overall survival rate following endoscopic submucosal dissection (ESD) for gastric tube cancer (GTC) using the Kaplan-Meier method. The 1-, 3-, and 5-year overall survival rates were 100%, 70.9%, and 70.9%, respectively.

The 1-, 3-, and 5-year overall survival rates were 100%, 70.9%, and 70.9%, respectively (Figure 3), with a median follow-up period of 35 months (range, 3-111 months). Five patients (27.8%) died of non-GTC disease, 2 died of infectious pneumonia unrelated to the GTC or ESD, 1 died of interstitial pneumonia, 1 died of colon cancer, and one died of renal failure. No GTC- or ESD-related deaths were observed during the study period. Of the 2 cases with SM2 invasion, lymphovascular invasion and positive vertical margins, one patient underwent an additional surgical resection of the reconstructed gastric tube with lymph node dissection. A residual lesion was detected in the resected specimen, but no lymph node metastases were observed. This patient is alive and in good condition 50 months after ESD with no evidence of recurrence. Another patient was followed with no additional surgery due to a comorbidity of interstitial pneumonia and low performance status; local recurrences were detected 2 months after ESD. This patient died of exacerbation of interstitial pneumonia 28 months after ESD, and no lymph node or distant metastases were observed in the remainder of the patient's life. In the 2 patients who underwent noncurative resection and who had only positive horizontal margins, no local recurrences were observed during the study period (Table 3). No metachronous GTC was diagnosed on follow-up endoscopy after ESD.

## 4. Discussion

In this study, we show that ESD for GTC was feasible and acceptable to enable *en bloc* resection and to prevent cancer-related death. However, even if an adverse event occurs, a serious treatment course may be possible because the surroundings encompass the mediastinum.

In terms of treatment outcomes, it was impossible to perform ESD in the retroflex position in 5 lesions because of the narrow working spaces. Nevertheless, we attempted to perform ESD in both the retroflex and straight positions in our study. Therefore, we are concerned that ESD for GTC is a technically demanding treatment. However, *en bloc* resection by ESD was possible in all cases in our study. In contrast, the complete *en bloc* resection rate and the curative resection rate of ESD were each 80%. Of the cases that did not receive complete *en bloc* and curative resection, 2 lesions were SM2-invasive cancers, and these lesions were endoscopically suspected to exhibit submucosal deep invasion before ESD. For these lesions, we performed ESD for total pathologic diagnosis because surgical resection of GTC is an invasive procedure with high morbidity and mortality [23]. Nonaka et al. [14] also reported that 13% of ESD for GTC cases involved lesions outside the ESD indication. The patients with noncomplete resection with positive horizontal margins had 3 lesions. A likely reason for positive horizontal margins was inaccurate endoscopic evaluation of the horizontal extent of GTC. In the reconstructed gastric tube, mucosal changes with severe inflammatory metaplastic gastritis were expected to be induced by various factors, such as *Helicobacter pylori* infection and the reflux of duodenal juice, including bile and blood flow disturbance; this is similar to what is observed in the remnant stomach after gastrectomy [24–26]. Mucosal changes in the gastric tube might be associated with an inaccurate evaluation of the horizontal extent. In this study, the interval between the esophagectomy and ESD was long (median period, 103 months) compared with other reports [11, 13, 16, 17]. Therefore, inflammation of the gastric tube mucosa may have been more severe.

In terms of long-term outcomes, the 1-, 3-, and 5-year overall survival rates were 100%, 70.9%, and 70.9%, respectively, but no GTC- or ESD-related deaths were observed. Of the 16 lesions that were removed by curative resection and the two lesions with positive horizontal margins that were removed by noncurative resection, no local recurrences, lymph node metastases, or distant metastases were observed. ESD prevented the death of these patients from GTC. In contrast, 2 cases of SM2 invasion were observed in this study. Additional surgery was performed for 1 patient who is still alive and in good health. Follow-up without additional surgery was performed for another patient. This patient died of exacerbation of a comorbidity and not from GTC; nevertheless, local recurrences were detected. Bamba et al. [12] reported that the rate of lymph node metastases in GTC with submucosal invasion was 4.8%. Further studies with large numbers of patients with GTC are necessary to confirm the clinical outcomes of ESD for GTC with submucosal invasion and the additional surgeries that are performed for these lesions.

In terms of adverse events, two serious events occurred in this study, including intraoperative perforation that required emergency surgery and pyothorax that required chest drainage. Previous studies have reported that the risk of intraoperative perforation in gastric ESD of the unresected stomach ranges from 1.2% to 5.2% [27]. In contrast, Nonaka et al. [14] reported that the risk of intraoperative perforation in ESD for GTC was 3.8%. In this study, intraoperative perforation occurred in 5% of the lesions, which was similar to the findings of previous reports. Several researchers have reported that almost all perforations in gastric ESD were successfully treated with conservative treatment using endoscopic clips to close the perforations [27, 28]. However, our case of intraoperative perforation required emergency surgery due to mediastinitis. The likely reason for the serious mediastinitis was refluxed gastric juice as well as refluxed duodenal juice, including bile that leaked to the mediastinum. This occurred because normal antireflux mechanisms involving the lower esophageal sphincter, angle of His, and phrenoesophageal ligament had been resected or disrupted by the esophagectomy [29]. In addition, negative intrathoracic pressure and positive intra-abdominal pressure might act together to promote duodenal juice reflux. Another likely reason was that the healing of the perforated portion was delayed due to insufficient blood supply to the gastric tube [30]. In two reports, delayed perforation after ESD for GTC occurred in 12.5% [11, 31] and 2.5% [14] of patients, respectively, due to reduced vascular circulation within the gastric tube. Therefore, more careful management of ESD for GTC is required to prevent intraoperative and delayed perforation. We experienced a case of pyothorax after ESD, which is a very rare adverse event [32]. The likely reason for this was thermal injury caused by electrocauterization during ESD, which then spread around the pleura because the dilated and tortuous gastric tube markedly protruded into the right thoracic cavity close to the pleura (Figure 2(c)). Miyagi et al. [31] reported the occurrence of precordial skin burn due to thermal injury by electrical coagulation as a complication of ESD for GTC in presternal reconstruction [31]. When ESD is performed for GTC using the presternal route or when the gastric tube protrudes into the thoracic cavity, more careful management is required to prevent thermal injury caused by electrocauterization.

This study has several limitations. First, it was a small study with a retrospective design that was conducted at a single institution. Second, only 1 lesion on suture line and no lesions at the anastomotic site of the esophagectomy were included in this study. Several researchers reported that the complete *en bloc* rate was low when lesions overlapped with a suture line and/or an anastomotic site in GTC and remnant stomach cancer [13, 15]. Third, the follow-up period was short in terms of the overall survival.

## 5. Conclusions

In conclusion, ESD for GTC was feasible and acceptable to enable *en bloc* resection and to prevent GTC-related death. However, ESD for GTC should be performed more carefully than common gastric ESD because serious adverse events specific to the gastric tube may occur.

## Figures and Tables

**Table 1:** Clinicopathological characteristics in 20 lesions in 18 patients with gastric tube cancers.

Age, median (range) (years)	72.5 (55-82)
Gender (male/female)	17/1
Pathological stage of esophageal cancer (0/I/II/III/IV/unknown) (*n*)	8/2/3/1/0/4
Interval between esophagectomy and ESD, median (range) (months)	108 (24-264)
Reconstruction route (retrosternal/posterior mediastinal) (*n*)	10/8
Food residue in the stomach on ESD (present/absent) (*n*)	4/14
Tumor location (upper/middle/lower) (*n*)	1/9/10
Involving the stump line (yes/no) (*n*)	1/19
Macroscopic type (0-I/0-IIa/0-IIc) (*n*)	2/5/13
Resected specimen diameter, median (range) (mm)	36.5 (23-76)
Tumor diameter, median (range) (mm)	16 (8-61)
Histological type (differentiated/undifferentiated) (*n*)	19/1
Depth of tumor invasion (M/SM1/SM2) (*n*)	18/0/2
Lymphatic invasion (*n*) (%)	2 (10)
Venous invasion (*n*) (%)	1 (5)
Horizontal margin positive (*n*) (%)	3 (15)
Vertical margin positive (*n*) (%)	2 (10)
Ulcer finding (absent/present) (*n*)	19/1

ESD: endoscopic submucosal dissection; M: mucosal cancer; SM1: minimally invasive submucosal cancer, invasion depth < 500 *μ*M from the muscularis mucosa; SM2: invasive submucosal cancer, invasion depth ≥ 500 *μ*M from the muscularis mucosa.

**Table 2:** Treatment outcomes.

Procedure time, median (range) (min)	87.5 (19-242)
Procedure in retroflex position (possible/impossible/unnecessary)	4/5/11
*En bloc* resection (*n*) (%)	20 (100)
Complete *en bloc* resection (*n*) (%)	16 (80)
Curative resection (*n*) (%)	16 (80)

**Table 3:** Noncurative resection cases.

Case	Age	Location	Procedure in retroflex position	Interval between esophagectomy and ESD (month)	Reason for noncurative resection	Additional treatment	Follow-up duration (months)	Recurrence	Vital status	Cause of death in fatal cases
1	73	Middle	Unnecessary	88	HM+	Follow-up	25	None	Dead	Infectious pneumonia
2	72	Lower	Unnecessary	264	HM+	Follow-up	33	None	Dead	Colon cancer
3	70	Middle	Necessary	113	SM2, VM+, Ly+, V+	Follow-up	28	Local recurrence	Dead	Interstitial pneumonia
4	75	Middle	Necessary	60	SM2, HM+, VM+, Ly+	Surgical resection	58	None	Alive	—

ESD: endoscopic submucosal dissection. Reasons for noncurative resection: HM+: horizontal margin positive; SM2: invasive submucosal cancer, invasion depth ≥ 500 *μ*M from the muscularis; VM+: vertical margin positive; Ly+: lymphatic invasion positive; V+: vascular invasion positive.

**Table 4:** Adverse events.

Total events (*n*) (%)	3 (16.7)
Postoperative bleeding	1 (5.6)
Intraoperative perforation	1 (5.6)
Pyothorax	1 (5.6)

## Data Availability

The data used to support the findings of this study are available from the corresponding author upon request.
